# A Study of the Relationship between Food Group Recommendations and Perceived Stress: Findings from Black Women in the Deep South

**DOI:** 10.1155/2015/203164

**Published:** 2015-03-04

**Authors:** Tiffany L. Carson, Renee Desmond, Sharonda Hardy, Sh'Nese Townsend, Jamy D. Ard, Karen Meneses, Edward E. Partridge, Monica L. Baskin

**Affiliations:** ^1^Division of Preventive Medicine, Department of Medicine, School of Medicine, University of Alabama at Birmingham, 1720 2nd Avenue South, Birmingham, AL 35294, USA; ^2^Department of Epidemiology and Prevention, Wake Forest School of Medicine, Wake Forest Baptist Medical Center, Winston Salem, NC 27157, USA; ^3^Department of Nursing, School of Nursing, University of Alabama at Birmingham, Birmingham, AL 35294, USA; ^4^Division of Gynecologic Oncology, Department of Obstetrics and Gynecology, School of Medicine, University of Alabama at Birmingham, Birmingham, AL 35294, USA

## Abstract

Black women in the Deep South experience excess morbidity/mortality from obesity-related diseases, which may be partially attributable to poor diet. One reason for poor dietary intake may be high stress, which has been associated with unhealthy diets in other groups. Limited data are available regarding dietary patterns of black women in the Deep South and to our knowledge no studies have been published exploring relationships between stress and dietary patterns among this group. This cross-sectional study explored the relationship between stress and adherence to food group recommendations among black women in the Deep South. Participants (*n* = 355) provided demographic, anthropometric, stress (PSS-10), and dietary (NCI ASA-24 hour recall) data. Participants were obese (BMI = 36.5 kg/m^2^) and reported moderate stress (PSS-10 score = 16) and minimal adherence to Dietary Guidelines for Americans food group recommendations (1/3 did not meet recommendations for any food group). Participants reporting higher stress had higher BMIs than those reporting lower stress. There was no observed relationship between stress and dietary intake in this sample. Based on these study findings, which are limited by potential misreporting of dietary intake and limited variability in stress measure outcomes, there is insufficient evidence to support a relationship between stress and dietary intake.

## 1. Introduction

The United States Department of Agriculture (USDA) and the Department of Health and Human Services (DHHS) jointly issue the Dietary Guidelines for Americans every 5 years [1, 2]. Based on these guidelines, initiatives like MyPyramid and more recently, MyPlate, were developed to remind individuals to make healthy food choices and to be physically active [1, 2]. Fruits (1.5 cups/day), vegetables (2.5 cups/day), whole grains (6 ounces/day), low-fat dairy (3 cups/day), and lean meats (5 ounces/day) comprise the list of USDA recommended food groups [1, 2]. Consumption of dietary patterns consistent with the recommended food groups has been associated with a lower incidence of cancer [3, 4], heart disease [5], and an overall reduction in mortality [6, 7]. Despite the potential benefits, adherence to recommended dietary guidelines is extremely low [8]. Nationally, 80–90% of individuals fail to consume the recommended amount for each food group [8].

A potential contributor to individuals failing to meet dietary guidelines is psychological stress defined as the extent to which an individual perceives that his or her demands exceed the individual's ability to cope [9]. Stress has been linked to eating behavior in previous research [10–12]. In a 2005 study of triethnic, low-income women, stress, along with several other psychosocial factors, was associated with a less healthful diet [12]. Black women, in particular, report high levels of psychological stress even after controlling for socioeconomic factors [13–16] and experience excess morbidity and mortality related to many diseases with diet-related risk factors such as obesity, hypertension, type 2 diabetes, and some cancers compared to white women [17–20]. When considering black women living in the Deep South, these health disparities are even greater. Black women living in the Deep South have a higher prevalence of obesity, cancer, stroke, and hypertension than black women living in other parts of the country [21]. While there is some data available regarding the dietary patterns of blacks [22, 23], it is limited in nature for blacks living in the Deep South, a region where dietary practices may still be largely influenced by historical (e.g., slavery) and sociocultural (e.g., family traditions, economic conditions, and cultural norms) factors unique to the region [24].

To our knowledge, there are no published studies that have examined whether stress may influence adherence to the Dietary Guidelines for Americans food group recommendations among black women living in the Deep South. The purpose of this study was to examine the relationship between perceived stress and adherence to food group recommendations among these black women. Secondary outcomes included primary macronutrient (e.g., total fat, carbohydrate, and protein) and energy intake. We hypothesized that higher perceived stress would be negatively associated with meeting dietary guidelines and overall healthy eating habits.

## 2. Subjects and Methods

### 2.1. Study Participants

Participants were recruited through an ongoing academic-community partnership between academic researchers and community partners in 20 counties across Alabama and Mississippi [25, 26]. Within each county, black women who were at least 20 years of age were recruited from April 2011 through August 2011 by a local paid staff person who lived either in or near the county of interest. Potential participants were recruited by local staff through word of mouth, social networks, and/or community institutions (e.g., churches, health departments, and schools/educational programs). All study-related protocols and questionnaires received approval from The University of Alabama at Birmingham (UAB) Institutional Review Board (IRB) for human subjects.

### 2.2. Data Collection and Study Measures

**Figure 1:**
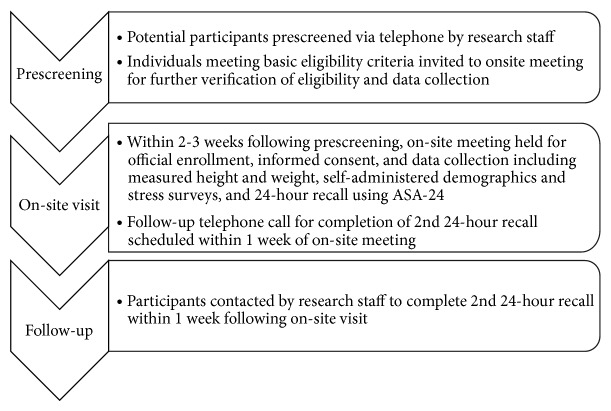
Study timeline.

The data collection schedule is illustrated in Figure 1. Participants completed all self-assessment surveys, height and weight measurements, and an interviewer-administered 24-hour dietary recall in a single on-site visit. While being on site, participants were also scheduled for a follow-up telephone call during which research staff completed the second 24-hour dietary recall within one week of the site visit.

#### 2.2.1. Demographics

A 13-item demographic data collection tool was used to gather the following: residence (rural, urban); age; education (less than high school, high school or equivalency certificate (GED), some college, college degree, or higher); employment (not employed, employed, and retired/disabled); household income (<$10,000, $10,000–$29,999, $30,000–$49,999, $50,000, or more); and history of selected health conditions (cancer, diabetes, heart disease/stroke, high blood pressure, and high cholesterol).

#### 2.2.2. Anthropometrics

Participants' weight and height were measured by trained research staff according to a standardized protocol. Weight was measured in indoor clothing, without shoes, on a calibrated digital scale (Seca 847, Hanover, MD). Height was measured using a calibrated stadiometer (Seca 217, Hanover, MD). BMI was calculated as weight (kg)/height (m^2^).

#### 2.2.3. Dietary Intake

Twenty-four hour dietary recalls were conducted using the National Cancer Institute's Automated Self-Administered 24-Hour Recall tool (NCI ASA-24), Beta Version [27, 28]. The NCI ASA-24 is a web-based tool for capturing 24-hour dietary intake drawn from the USDA's Automated Multiple-Pass Method. This tool uses the “gold standard” multiple-pass methodology. This web-based program allows researchers to enter dietary recall data via a respondent portal that is linked to a researcher site where data are saved for future download. Typically, the NCI ASA-24 employs Computer-Assisted Self-Interviewing (CASI) methodology to guide the respondent through multiple steps of recalls including meal-based quick list, meal gap review, detail pass (included quantity consumed), forgotten foods, and final review. However, due to both reading and computer literacy issues within our targeted population, research staff performed the 24-hour recalls in a one-on-one interviewer format. Data output includes a summary of respondent quick list and nutrients as well as MyPyramid equivalents from reported food and beverages. Two scheduled recalls were conducted: one weekday and one weekend day to better capture typical intake patterns. The rationale for two recalls was based on pilot testing with a similar population as the study target group. Pilot participants suggested that our target audience would likely complete one recall and perhaps a second one, but they suggested that beyond that, people like them would not be interested as they may view this as “getting too much in their business” particularly since this was not a nutrition intervention study. While 3 recalls have been suggested as the optimal number of recalls [29], it has also been noted that factors such as demographics (e.g., race, geographic region) and method of data collection (e.g., self-administered versus interview) should be taken into account when making final decisions. Resnicow et al. conducted a study in African American adults in the Deep South in which dietary carotenoid values for both 1 and 3 days of 24-hr recalls were estimated using dietary recall software and compared with measured serum carotenoid values. This comparison confirmed the validity of either 1 or 3 days of dietary recalls [30]. Based on Resnicow's work, as well as our pilot testing of the feasibility of collection, we chose to capture 2 recall days (1 weekday, 1 weekend day), both interviewer administered, with 1 in-person and 1 over the phone. Trained research staff conducted one recall in-person and a second recall over the telephone. The primary dietary outcome of interest was whether or not participants met the recommended number of servings for USDA recommended food groups: fruits, vegetables, whole grains, low-fat dairy, and lean meats. Secondary dietary outcomes of interest were overall energy and primary macronutrient intakes (e.g., fat, carbohydrate, and protein).

#### 2.2.4. Perceived Stress Scale-10 (PSS-10)

Participants completed the PSS-10, a ten-item scale used to assess the degree to which situations in one's life are appraised as stressful. The PSS-10 has been found to have adequate internal test-retest reliability (Cronbach's alpha = .88) and is positively correlated with a variety of self-report and behavioral indices of stress in adult populations [31]. The PSS-10 is not diagnostic in nature and cut-points have not been established, although there are population norms for comparison (*x*_women_ = 13.7; *x*_blacks_ = 14.7) [32]. Scores can be used to infer relative stress levels or within-group comparisons, with higher scores on the PSS-10 indicating greater perceived stress (possible range 0–40). For the stratified analysis conducted in this study, we performed a median-split to categorize participants into categories relative to one another (lower stress versus higher stress).

### 2.3. Statistical Analysis

Descriptive statistics were calculated as means for continuous variables including age, BMI, calories, and macronutrients; median for PSS-10 score due to nonnormal distribution; and frequencies for categorical variables including residence, education, employment, household income, and food group recommendation adherence. Unadjusted analyses to examine between-groups differences and associations included *t*-tests for continuous variables, chi-square tests for categorical variables, and Pearson correlations to test for associations between continuous variables. The relationship between PSS-10 score as the predictor and adherence to food group recommendations (yes or no for the following groups: meets fruits; meets vegetables; meets dairy; meets grains; meets meats and beans; and meets all groups) was examined with logistic regression controlling for age in years, total energy intake in kcal, BMI (kg/m^2^), and income (referent group = <$10,000, $10,000–$29,999, $30,000–$49,999, and >$50,000). Similarly, the relationship between PSS-10 score as the predictor and macronutrient intakes (protein, fat, and carbohydrates in grams) was examined with linear regression models controlling for age, energy, BMI, and income; a similar model (excluding energy as a control variable) examined the relationship between PSS-10 score and total calories (kcal). Similar regression models that included stress as a categorical variable (lower stress versus higher stress) based on a median split were also conducted.

## 3. Results

Participant characteristics are detailed in Table 1. Participants were 355 obese black women with a mean BMI and age of 36.5 kg/m^2^ and 49.8 years (range 20–86 years), respectively. The majority of participants reported some education beyond high school; however, only about half were employed, and 63% reported an annual household income of less than $30,000. Nearly 64% of participants reported at least one health condition that has been previously associated with diet in the literature including a current or previous diagnosis of cancer (9.3%), diabetes (28.2%), heart disease/stroke (7.6%), hypertension (63.7%), or high cholesterol (34.6%). For this study, the presence of one of the aforementioned health conditions was not associated with dietary intake and did not influence the relationship between stress and diet (data not shown). Overall, participants reported a mean PSS-10 score of 15.3 ± 6.8 (median = 16; range 0–35; Cronbach's *α* = 0.82). After being stratified as higher stress and lower stress based on the median score (i.e., those with PSS-10 ≥ 16 were categorized as higher stress; those with PSS-10 < 16 were categorized as lower stress), differences in sociodemographic factors emerged. Participants in the higher stress group were significantly younger (*P* < 0.001) and had a significantly higher BMI (*P* = 0.05) than those in the lower stress group. The lower stress group was more educated and more likely to be not currently working compared to individuals in the higher stress group (Table 1).

Overall, participants' adherence to the Dietary Guidelines for Americans food group recommendations was minimal. Nearly one-third of the sample did not meet the recommended intake for any of the food group categories. Another 35% met the recommended intake for only 1 category, while the proportion of remaining participants who met recommendations for 2, 3, or 4 categories was 25%, 8%, and 1%, respectively. The proportion of women who did not meet each of the food group recommendations did not differ by stress level. Overall mean values for calorie, total fat, carbohydrate, and protein intake were 1453.0 kcal, 57.7 g, 172.9 g, and 66.5 g, respectively. Unadjusted and adjusted analyses, controlling for age, BMI, energy, and income, indicated no significant relationships between PSS-10 score and meeting recommended food groups or primary macronutrient intake (Table 2). There were no differences in macronutrient or energy intake by stress group (calories: 1443 versus 1465 kcal, *P* = 0.77; total fat: 57 versus 58 g, *P* = 0.90; carbohydrate: 171 versus 175 g, *P* = 0.70; and protein 66 versus 66 g, *P* = 0.86 for lower stress versus higher stress).

## 4. Discussion

Our findings demonstrate inadequate intake across all recommended food groups for the majority of our sample of black women living in the Deep South. Failure to adopt a dietary pattern consistent with USDA guidelines and recommendations may partially explain the health disparities seen among black women living in the Deep South. For instance, nearly 64% of women in our sample reported hypertension compared to a national average of 45% for black women in the US [33]. Similarly, the prevalence of diabetes and high cholesterol among participants in the current study was higher than the national averages for black women (diabetes: 23% versus 13%; high cholesterol: 35% versus 28%) [34]. Research has shown that dietary patterns consistent with USDA guidelines can reduce morbidity and mortality from leading chronic conditions [3–7] and it is critical to continue to conduct rigorous research to identify potential barriers (e.g., stress, availability, economic, and environment) and facilitators (e.g., education, social support, and health promoting interventions) for meeting the recommendations.

The low level of adherence to recommended food group intake is consistent with previous studies, particularly for impoverished individuals, although the proportion of nonadherent individuals is even higher among the population in the current study. For example, George et al. examined compliance with dietary guidelines among a low-income sample of postpartum women and found that, for most food groups, one-quarter or less of participants were compliant [12].

Though higher stressed women were significantly heavier than the lower stress group, we did not observe a significant relationship between dietary intake and stress level among participants. This lack of relationship between food group intake and stress level is inconsistent with previous research [35] in other populations. The current findings may be explained by a utilization of coping strategies outside of food, for example, spirituality [36], which may mitigate the impact of stress on eating behaviors for black women in the Deep South participants. Despite the fact that we observed no direct relationship between stress and dietary intake in our sample, other modifiable environmental and sociocultural factors including coping strategies, cost/availability of food, cultural food practices, and nutrition-related knowledge may contribute to food choices and should be further studied.

*Limitations*. The findings of this study are limited by potential recall bias or misreporting of dietary intake data. Prior research has suggested that women may underreport energy intake by 16–20%. Though we are unable to determine the true amount of underestimation among participants in the current study, if we conservatively assume that participants' energy intake should be at least equal to their basal metabolic rate, it would suggest that 67% of our study sample underreported their energy intake on the days of recall. We used the Harris-Benedict equation to estimate basal energy needs, and on average, the underestimation was 33% below basal metabolic rate, ranging from negligible to greater than 50% for some. A study by Subar et al. reported that only 7% of women underreported both energy intake and protein suggesting a bias towards greater underreporting of fat, carbohydrate, and alcohol [37]. This underreporting of energy and intake of macronutrients that have been linked to stress in other larger studies [38, 39] may have biased our ability to detect any relationship between dietary intake and stress in our sample. Sensitivity analyses using the assumptions about underestimation described above also revealed that women who underestimated their energy intake had significantly higher BMIs than women who did not underestimate energy intake. This finding taken together with the fact that women who reported higher stress levels had higher BMIs than those with lower stress increases the likelihood that potential relationships between diet and stress were biased towards the null due to misreporting of dietary data. Another limitation is that the overall stress level of the sample population was not high and showed limited variability, which is contrary to some previous reports [13, 14], but may be explained by a phenomenon known as the Superwoman Schema adopted by some black women [40]. The Superwoman Schema role includes an “obligation to manifest an image of strength and minimize reports of stress” [40]. Therefore, decreased endorsement of stress and/or misreporting of dietary intake may have limited our ability to detect significant relationships between stress and dietary intake. Additionally, particular subgroups exhibiting high stress may have a unique dietary pattern that was not observable in this study group. Another limitation of the study findings is that MyPyramid equivalents, which are now considered antiquated by some due to the introduction of MyPlate, were used in the data analysis. However, at the time of study development and inception, MyPyramid was the standard endorsed by the USDA and guided the development of the study protocol. Though MyPyramid and MyPlate differ visually, the information about diet content and amount remains the same. Therefore, the proportion of participants who met food group recommendations would not change based upon which food group guide was utilized in the study protocol. Finally, the original study did not include any assessment of physical activity. The academic-community partnership that conducted this research was responsive to the input of the community which wanted to focus efforts on the diet and nutrition of community members. Thus, physical activity, which may have influenced the relationship between stress, dietary intake, and BMI in this sample, was not measured. Research has shown that higher stressed individuals are more likely to be sedentary [41] and that physical activity can reduce stress [42]. Additional research has also shown that, among a sample of black women, those who were more physically active also ate more fruits and vegetables [43]. Since physical activity is related to both stress level and dietary intake, participants' physical activity level may have mediated the relationship between stress and dietary intake and may also explain why those in the higher stress strata had a higher mean BMI.

## 5. Conclusions

Among this group of black women living in the Deep South, adherence to the food groups outlined in the dietary guidelines is minimal but does not appear to be linked to higher psychological stress. Though previous research has suggested that stress is a barrier to meeting recommendations, the current study suggests that more appropriate measurements of key variables (e.g., stress, dietary intake) may be needed to adequately investigate relationships between stress and dietary intake. Additionally, other factors that may contribute to dietary behaviors may need to be considered in this unique population. Future research can incorporate qualitative methods and analyses to further explore why individuals in our target population, on average, fail to meet dietary recommendations. Additional exploration of the relationship between stress, diet, and adherence to dietary guidelines, including utilization of more objective measures and a more in-depth consideration of coping and physical activity, is warranted in this group.

## Figures and Tables

**Table 1:** Demographics of black women in Deep South dietary study stratified by stress scores.

	Total	Lower stress (PSS < 16)	Higher stress (PSS ≥ 16)	*P* value
	(*n* = 355)	(*n* = 192)	(*n* = 163)	
	Mean ± sd	Mean ± sd	Mean ± sd	
Age	49.8 ± 15.3	52.8 ± 14.2	46.2 ± 15.7	<0.001
BMI (kg/m^2^)	36.5 ± 8.6	35.7 ± 8.4	37.5 ± 8.6	0.05

	*N* (%)	*N* (%)	*N* (%)	

Residence				
Rural	296 (83.4)	162 (84.3)	134 (82.1)	0.58
Urban	59 (16.6)	30 (15.7)	29 (17.8)	
Age category				
20–39	102 (28.7)	40 (20.8)	62 (38.0)	<0.001
40–59	148 (41.7)	82 (42.7)	66 (40.5)	
60+	105 (29.6)	70 (36.5)	35 (21.5)	
Education				
<HS diploma	38 (10.7)	16 (8.3)	22 (13.5)	0.05
HS diploma/GED	63 (17.7)	29 (15.1)	34 (20.9)	
Some college	99 (27.9)	52 (27.1)	47 (28.8)	
College degree+	155 (43.7)	95 (49.5)	60 (36.8)	
Employment				
Employed	173 (48.7)	90 (46.9)	83 (50.9)	0.02
Unemployed	35 (9.9)	14 (7.3)	21 (12.9)	
Retired/disabled	131 (36.9)	84 (43.8)	47 (28.8)	
Student	16 (4.5)	4 (2.0)	12 (7.4)	
Household income				
<$10,000	95 (26.8)	44 (22.9)	51 (31.3)	0.09
$10,000–$29,999	125 (35.2)	64 (33.3)	61 (37.4)	
$30,000–$49,999	80 (22.5)	46 (24.0)	34 (20.9)	
$50,000+	49 (13.8)	33 (17.2)	16 (9.8)	
Did not repot	6 (1.7)	5 (2.6)	1 (0.6)	

**Table 2:** Relationship between higher stress and dietary intake among black women in the rural Deep South.

USDA recommended food groups	OR (95% CI)^a^	OR (95% CI)^a,b^
Fruit	1.27 (0.81, 2.00)	1.04 (0.64, 1.71)
Vegetables	1.38 (0.75, 2.52)	0.98 (0.51, 1.88)
Dairy	1.71 (0.31, 9.47)	1.55 (0.25, 9.43)
Grains	0.69 (0.43, 1.10)	0.67 (0.41, 1.09)
Meat and beans	0.79 (0.51, 1.21)	0.79 (0.50, 1.25)

Mean intake	*B* (se)^c^	*B* (se)^b,c^

Energy (kcal)	3.23 (5.40)	−2.18 (5.80)
Protein (g)	0.04 (0.28)	−0.17 (0.30)
Carbohydrate (g)	0.62 (0.61)	0.29 (0.38)
Fat (g)	0.02 (0.29)	−0.11 (0.12)

^a^Logistic regression modeling stress score as a predictor meeting the following dietary requirements: fruit = 1.5 cups/day, vegetables = 2.5 cups/day, dairy = 3 cups/day, and grains = 6 ounces/day, 5 ounces/day; a cup equivalent is equal to 1 cup of fruit or fruit juice, 1 cup of raw or cooked vegetables or vegetable juice, and 1 cup of milk; ounce equivalents: an ounce-equivalent of grains is equal to 1 slice of bread and an ounce-equivalent of meat and beans is equal to 1 ounce of cooked meat, poultry, or fish. 1 ounce = 28 g; ^b^controlled for age in years, income (<$10,000, $10,000–$29,999, $30,000–$49,999, and $50,000+), BMI (kg/m^2^), and total energy (except when energy is outcome variable). ^c^Linear regression modeling stress score as a predictor of mean intake of energy and macronutrients.
